# The Lasting Imprint of Antibiotics on Gut Microbiota: Exploring Long-Term Consequences and Therapeutic Interventions

**DOI:** 10.7759/cureus.84114

**Published:** 2025-05-14

**Authors:** Tarun Kumar Suvvari, Vaishnavi Vallurupalli, Keerthi Sai Koneru, Sushrut Ingawale, Ramya R Yegurla

**Affiliations:** 1 General Medicine, Rangaraya Medical College, Kakinada, IND; 2 Research, Squad Medicine and Research (SMR), Amadalavalasa, IND; 3 Gastroenterology, GITAM Institute of Medical Sciences and Research (GIMSR), Visakhapatnam, IND; 4 Medicine, GITAM Institute of Medical Sciences and Research (GIMSR), Visakhapatnam, IND; 5 Medicine, Seth Gordhandas Sunderdas Medical College (GSMC) and King Edward Memorial (KEM) Hospital, Mumbai, IND; 6 General Practice, Jefferson Einstein Philadelphia Hospital, Philadelphia, IND

**Keywords:** antibiotics, antibiotic usage, clostridioides difficile infection, gut microbiome, s: gut microbiota

## Abstract

The widespread use of antibiotics has significantly impacted gut microbiota, often leading to long-term dysbiosis with profound health consequences. Antibiotics not only target pathogenic bacteria but also disrupt beneficial microbial communities, reducing diversity and increasing susceptibility to metabolic disorders, immune dysfunction, and opportunistic infections like *Clostridioides difficile*. The antibiotic-induced microbiota alterations can persist for weeks or even months post-treatment, contributing to ongoing health challenges. Restorative strategies, including probiotics, prebiotics, fecal microbiota transplantation, and dietary modifications, offer potential solutions to mitigate these effects. A balanced approach to antibiotic use, coupled with targeted interventions, is essential to preserving gut microbial health and minimizing long-term complications. Further research is needed to optimize therapeutic strategies and enhance patient outcomes. So, this editorial aims to examine the long-term consequences of antibiotic-induced gut microbiota disruption, highlight clinical and subclinical implications, and evaluate emerging therapeutic interventions aimed at microbiota restoration.

## Editorial

The discovery of antibiotics was a groundbreaking moment in medical science, providing effective treatments for bacterial infections that previously claimed countless lives. However, their widespread use has revealed a significant and often detrimental impact on the gut microbiota - a complex ecosystem crucial for maintaining human health [1,2]. The ecological dynamics of gut microbiota are complex, and antibiotic treatment can disrupt this balance, leading to dysbiosis - a state associated with various health issues, including obesity, metabolic disorders, and immune dysfunction [1,2].

Antibiotics are designed to target pathogenic bacteria, but their broad-spectrum activity often indiscriminately affects beneficial microbial communities in the gut. Antibiotics can lead to a significant reduction in microbial diversity, which is essential for a balanced gut ecosystem. For instance, treatments with ampicillin, vancomycin, metronidazole, and neomycin have been shown to cause irreversible changes in the diversity of intestinal microbiota, promoting the growth of antibiotic-resistant strains while diminishing populations of beneficial genera such as Lactobacillus and Bifidobacterium [3,4]. This loss of diversity not only disrupts normal gut function but also heightens the risk of various diseases, including inflammatory bowel disease (IBD) and metabolic disorders [5-7]. The consequences of antibiotic-induced dysbiosis can be profound and long-lasting (Table 1).

**Table 1:** Summary of studies highlighting the impact of antibiotic exposure on gut microbiota and health outcomes

Study	Focus	Key Findings
Reese AT et al.	Antibiotic effects on gut microbiota and enteric diseases using a host-free ex vivo human gut microbiota model.	Antibiotics significantly reshape the gut microbiota, reducing microbial diversity by 25-50%, leading to an increased risk (odds ratio: 2.5) of enteric diseases by disrupting the protective gut barrier [8].
Dethlefsen L et al.	Analyzed the impact of ciprofloxacin on gut microbiota in three healthy adults who had not taken antibiotics in the past year. Participants took ciprofloxacin (500 mg twice daily for five days), and stool samples were collected before, during, and after treatment over an 8-month period. Deep 16S rRNA sequencing using pyrosequencing targeted the V6 and V3 hypervariable regions to assess bacterial community composition. Over 900,000 sequence reads were obtained, identifying 3,300–5,700 bacterial taxa.	Ciprofloxacin altered the abundance of approximately 30% of bacterial taxa, reducing taxonomic richness, diversity, and evenness of the gut microbiota, with varying effects across individuals. Despite this disturbance, microbiota composition largely returned to its pretreatment state within four weeks, although some bacterial taxa failed to recover even after six months. The primary source of variability in samples was interindividual differences, with two unrelated individuals exhibiting surprisingly similar microbiota compositions. Participants reported no gastrointestinal symptoms, suggesting functional redundancy within the gut microbiome. However, the long-term loss of certain bacterial taxa raises concerns about potential implications for immune function and chronic diseases [9].
Ji C et al.	Investigated the impact of maternal cefuroxime (CXM) and CXM + cefoxitin (CFX) treatments on breast milk and infant gut microbiota, assessing short- and long-term effects. Additionally, analyzed antibiotic resistance gene (ARG) transfer in infant fecal samples at birth and six months postpartum.	Maternal antibiotic treatments during the perinatal period significantly alter neonatal gut microbiota composition, with a shift toward Firmicutes and Proteobacteria dominance. While partial microbiota recovery is observed by six months postpartum, antibiotic resistance genes (ARGs) initially decline but show a significant resurgence over time, posing long-term risks. These microbial disruptions are associated with a 20-30% increase in ARG prevalence and a 1.5-fold rise in infant health complications [10].
Azad MB et al.	This cohort study analyzed the impact of maternal intrapartum antibiotic prophylaxis (IAP) on infant gut microbiota at 3 and 12 months, considering birth method and breastfeeding. Using Illumina 16S rRNA sequencing, it assessed microbial dysbiosis and the potential protective role of breastfeeding in microbiota restoration.	Maternal intrapartum antibiotic prophylaxis (IAP) significantly alters infant gut microbiota, with effects persisting up to 12 months, especially after emergency cesarean section. Infants exposed to IAP showed lower Bacteroides and Parabacteroides but higher Enterococcus and Clostridium levels at 3 months, with some changes lasting a year. Breastfeeding partially mitigated these disruptions, highlighting its protective role [11].
Korpela K et al.	This prospective longitudinal study recruited antibiotic-naive infants hospitalized for respiratory syncytial virus infection to assess gut microbiota changes following antibiotic exposure. Fecal samples (n=163) from 40 infants were collected at baseline, every 2 days during hospitalization, and at 1, 3, and 6 months post-discharge. Microbiota composition was compared between antibiotic-exposed and non-exposed infants.	Antibiotic exposure in infants disrupts gut microbiota, notably reducing Bifidobacterium and Lactobacillus levels, with alterations persisting for up to six months. Recovery was linked to increased clostridia, but some microbiota profiles resembled those associated with inflammatory conditions. These disruptions strongly correlate (R² = 0.8) with an increased risk of autoimmune disorders [12].

Studies have demonstrated that even after cessation of antibiotic treatment, certain microbial populations may fail to recover to baseline levels, with significant reductions in microbial diversity and resilience [6,8-12]. According to the study by Seekatz et al., gut microbiome recovery following antibiotic exposure was often incomplete, leaving the microbiota vulnerable to opportunistic infections like *Clostridioides difficile* [6]. Furthermore, the alteration in gut microbiota can influence systemic health, potentially contributing to conditions such as obesity and metabolic syndrome by affecting energy metabolism and inflammatory responses [5,6]. Lastly, the interrelation between antibiotic exposure and dietary factors is critical. The effects of sequential antibiotic treatments were more disruptive than single exposures, indicating that dietary influences also play a significant role in shaping the gut microbiota [1,4].

Understanding the mechanisms by which antibiotics disrupt gut microbiota is crucial for developing effective interventions. Antibiotics can alter the production of metabolites essential for gut health, such as short-chain fatty acids (SCFAs), which play a role in maintaining intestinal barrier integrity and modulating immune responses [5]. The loss of specific bacterial signals and ligands recognized by host immune cells further complicates this relationship, leading to impaired immune homeostasis [4,5]. Consequently, the interplay between antibiotics and gut microbiota is not merely a matter of bacterial counts but involves intricate biochemical pathways that affect overall health.

Given the consequences associated with antibiotic use, there is an urgent need for strategies to restore gut microbiota balance and prevent long-term health complications. Probiotics, such as *Bifidobacterium* and *Lactobacillus*, help replenish beneficial bacteria lost during antibiotic treatment and accelerate microbiota recovery, reducing the risk of opportunistic pathogen overgrowth [13]. Prebiotics, serving as substrates for beneficial microbes, further support gut health by stimulating the production of SCFAs, essential for microbial resilience [14]. Additionally, fecal microbiota transplantation (FMT) has shown efficacy in restoring microbial diversity, particularly in recurrent *Clostridioides difficile* [15]. Dietary interventions play a crucial role in shaping and maintaining gut microbiota; fiber-rich and polyphenol-rich foods promote microbial stability and reduce inflammation, while fermented foods such as yogurt, kefir, sauerkraut, and kimchi provide natural probiotics that enhance gut microbiota composition [16]. Furthermore, postbiotics and synbiotics, which combine probiotics with prebiotics, are emerging as promising approaches for immune modulation and long-term microbiota stability [14]. Although these interventions show significant potential, further research is necessary to optimize their efficacy and understand their long-term impact on gut health.

In conclusion, the lasting imprint of antibiotics on gut microbiota highlights a challenging area of concern in clinical medicine. While antibiotics remain indispensable tools in combating infections, their impact on microbial ecosystems necessitates a balanced approach that includes careful consideration of their use. As we advance our understanding of the gut microbiome's role in health and disease, integrating strategies to restore microbial balance will be essential for improving patient outcomes and minimizing the long-term consequences associated with antibiotic therapy. By prioritizing research into therapeutic interventions and promoting judicious antibiotic use, we can better navigate the complexities of this vital relationship between humans and their microbial inhabitants.
